# A Comparison between Antibacterial Activity of Propolis and Aloe vera on *Enterococcus faecalis* (an *In Vitro* Study)

**Published:** 2013

**Authors:** Maryam Ehsani, Mahmood Amin Marashi, Ebrahim Zabihi, Maryam Issazadeh, Soraya Khafri

**Affiliations:** 1 *Dental Materials Research Center, School of Dentistry, Babol University of Medical Sciences, Babol, Iran.*; 2 *Department of Bacteriology and Virology, School of Medicine, Alborz University of Medical Sciences, Karaj, Iran.*; 3 *Cellular and Molecular Biology Research Center (CMBRC), Babol University of Medical Sciences, Babol, Iran.*; 4 *Department of Pharmacology & Physiology, School of Medicine, Babol University of Medical Sciences, Babol, Iran.*; 5 *Department of Social Medicine, School of Medicine, Babol University of Medical Sciences, Babol, Iran.*

**Keywords:** Chlorhexidine, root canal, antiseptic, *S. aureus*, *S. mutans*

## Abstract

Removing the bacteria, including *Enterococcus faecalis,* from the root canal is one of the important aims in endodontic treatment.We aimed to compare the antibacterial activity of Chlorhexidine with two natural drugs. The antibacterial activities of three different propolis extracts (alcohol concentrations: 0, 15, 40%) and Aloe vera gel on *E. faecalis* were compared using three methods: disk diffusion, microdilution and direct contact test. In addition to the above bacterium, the Aloe vera gel effect on *Staphylococcus aureus *and* Streptococcus mutans *was evaluated. Disk diffusion test revealed that propolis ethanolic extracts (the alcohol concentration of 15 and 40%) and Aloe vera gel have antibacterial activities but aqueous extract of propolis did not show any effect in this test. The MICs for propolis ethanolic extracts, Aloe vera gel and aqueous extract of propolis (0% alcohol) were 313 µg/ml, 750 µg/ml, 2250 µg/ml, and ≥ 500 µg/ml respectively, much higher than the Chlorhexidine one. In direct contact test, contrary to Aloe vera, all three propolis extracts showed antibacterial effects on *E. faecalis. *The Aloe vera gel also showed significant antibacterial effect on *S.aureus* and *S.mutans*. The hydroalcoholic extracts of propolis and Aloe vera gel had antibacterial effects on *E. faecalis*, however, propolis is more potent than Aloe vera. The antibacterial effect of Aloe vera on *S. aureus* and *S. mutans* is low (MIC ≥ 2250 µg/ml). Appropriate concentrations of alcoholic extracts of propolis and some fractions of Aloe vera gel might be good choices for disinfecting the root canal in endodontic treatments.

One of the main goals in endodontic treatments is removing the bacteria from the root canal system. Although chemo - mechanical preparation of root canal is able to decrease the bacterial load, the resistant microorganisms usually remain in the canal space even after the instrumentation and washing processes. The main reasons behind this contamination are: the complex anatomy of pulp system, existence of the secondary canals, and ability of microorganisms to survive in harsh conditions (1-2). *E. faecalis *is an anaerobic gram-positive bacterium which is found in periapical lesions. It is able to attack dentinal tubules and easily copes with hard condition of root canal which make it a resistant microorganisms (3). Some studies on root treated teeth have shown that *E. faecalis* bacteria are prevalent up to 77% in the periradicular lesions. In fact, the involvement of this bacterium in root canal treatment failure is more likely than the primary endodontic lesions (4). Sodium hypochlorite has been used as an intracanal irrigant, however, due to its adverse effects including damage to tissues and inducing emph-ysema, its used has been restricted. Chlorhexidine 2% solution is used as an intracanal irrigant with antibacterial properties and great ability to disinfect the dentinal tubules against *E. faecalis*, however its use has been restricted due to: discoloration of the teeth and tongue, decreasing the sense of taste, irritation of oral mucosa and mouth dryness. Nowadays, due to its antibacterial properties, calcium hydroxide is highly used as the intracanal medication. But again, because of its high pH, this subtance is so toxic to the tissues which can lead to chronic inflammation and cell necrosis (5-6). Because of the cytotoxicity induced by common intracanal drugs, their inability to remove some bacteria from the dentinal tubules, and the microorganisms’ resistance phenomenon, looking for new intracanal drugs especially among natural resources are highly recommended (7). 

Propolis is a dense yellow-brown resin-like material which its solubility is low in water, but high in ethanol (8). This material is made from resin, bud and other parts of the plants by bees. It is used for protecting the hive against the outside pollutions and blocking the slots and cracks. Propolis has antibacterial, antifungal, antiviral, antiinflammation, antioxidant and anti-tumor effects (8-9) and many applications for this substance in dentistry has been recently reported (7). Aloe vera, along with other 360 species, belongs to liliaceae family. This plant can grow in hot and dry weather due to its high capacity in maintaining water. Aloe vera has antibacterial, anti-fungal, antivirus, antiinflammation, and anti-tumor properties which make it useful in broadrange of ailments including: arthritis, asthma, gastrointestinal diseases, and skin problems (e.g. psoriasis, burning and wounds).

In dentistry, Aloe vera has been used in recurrent aphthous ulcers, alveolar osteitis, and lichen planus lesions (10-12).

The aim of this study was to determine the antibacterial potency of Aloe vera compared to propolis and Chlorhexidine. Also, the effect of ethanol concentration on antibacterial activity of hydroalcoholic extracts of propolis was investigated.

## Materials and Methods

**Propolis quality control assays**

About 150 grams of propolis was freshly collected from Amirkola’s (Mazadaran-Iran) honey bees’ nests during the 2012 winter. Standard microbiological and chemical assays were performed on the sample by Suren Tak Toos Lab. Co. (Mashhad-Iran).

**Propolis hydroalcoholic extraction**

Propolis was dispersed in absolute ethanol (500 mg in 50 ml) at 37ºC using magnet stirring for 1.5 hours. The obtained opaque yellow liquid passed through filter (Whatman#1) and centrifuged at 22ºC for 10 minutes (800 g). The clear supernatant was diluted with appropriate amounts of sterile distilled water to give ethanol concentration of either 15%. or 40%. To make aqueous extract, propolis was dispersed in sterile distilled water (500 mg in 50 ml) at 22ºC using magnet stirring for 4 hours. The obtained opaque liquid was filtered and centrifuged at 22ºC for 10 minutes (800g). These extracts were kept in the fridge (less than 1 week) and by warming up to 37ºC any precipitate was dissolved before use.

**Aloe vera physicochemical analyses**

Aloe vera gel was kindly gifted by Barij Essence (Kashan-Iran). Standard physicochemical assays including carbohydrates content, dry substance, ash weight, and capillary viscometry were performed.

**The test microorganisms**

The sample of standard strains of *E. faecalis* PTCC 1394, *S. mutans* ATCC 1601 and *S. aureus* ATCC 25923, were obtained from the Scientific-Industrial Research Center of Asre-Enghelab (Tehran-Iran) and were inoculated in Brain Heart Infusion (BHI) culture medium.

**Disk diffusion test**

The method of Kirby-Bauer disk-diffusion was performed for this assay. Briefly sterile paper disks (6.4 mm) were soaked in the test material solutions for 10 minutes. Ethanol (15, 40%) and distilled water were used as negative control. The impregnated paper disks were placed on the surface of blood agar culture plates previously inoculated by the test microorganism (*E. faecalis, S. mutans, S. aureous). *The inhibition zone was measured for each test material.

**Direct contact test**

The test material solutions (500 µL each) were dried on the bottom of a 24-well plate. Then 50 µL of the test bacterial suspension (1.5×10^7 ^CFU/ml) was poured into each well and left to dry in a laminar airflow. After that, 500 µL of BHI was added to each well and the plate was incubated at 37ºC. After 24 hours, the colony count of 5 µL of each well’s solution was measured.

**The microdilution test**

Broth microdilution test was performed as described in M27-A2 (CLSI) with minor modi-fications. The test material solutions was firstly diluted 50:50 in 2X BHI medium then serial dilutions were made using (100 µL) 1X BHI in each well, then 10 µL of microbial suspension (1.5×10^7 ^CFU/ml) was added. After 24 hours incubating at 37ºC, the last well without opacity was considered as minimum inhibitory concen-tration (MIC). The well with lowest concentration of the tested material, which could not lead to microbial growth (99.9% inhibition) after inocu-lating the blood agar plate, was considered as the minimum bactericidal concentration (MBC). Also the microdilution test was performed on Aloe vera using two additional microorganisms (*S.aureus* and *S.mutans*).

**Statistical analyses**

The data are presented as mean±SD and analyzed by ANOVA. In case of significance, the multi fold Scheffe comparisons and t-test were used for two by two comparisons. P < 0.05 was consid-ered significant.

## Results

The antibacterial activity of propolis hydroalcoholic extracts (with 0, 15, 40% ethanol), Aloe vera gel, and Chlorhexidine 2% on *E. faecalis *bacteria are compared using three methods: disk diffusion, direct contact and microdilution. In regards to Aleo vera, disk diffusion and micro-dilution tests, have been performed usingtwo additional bacteria (*S. aureus, S. mutans*) to investigate more its antimicrobial spectrum.

**Propolis and Aloe vera quality control assays**

The results of some quality control tests on propolis are shown in table 1. The physicochemical analysis data of Aloe vera are shown in table 2.

**Disk diffusion test**

**Fig 1:**
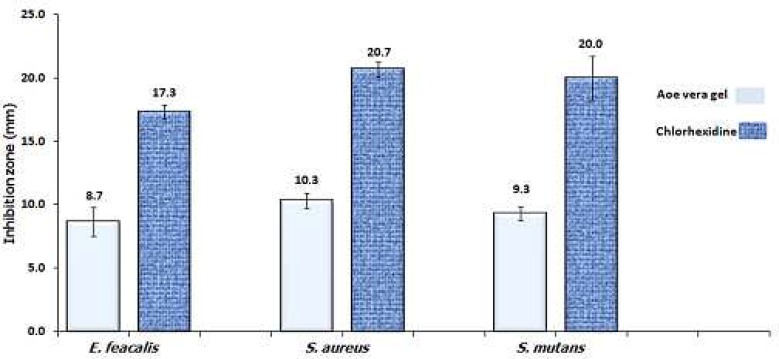
Growth inhibition zone (mean ±SD) induced by different propolis hydroalcoholic extracts (with 0, 15, 40% ethanol), Aloe vera gel and chlorohexidine 2% in the method of disk diffusion with *E. faecalis*

**Fig 2:**
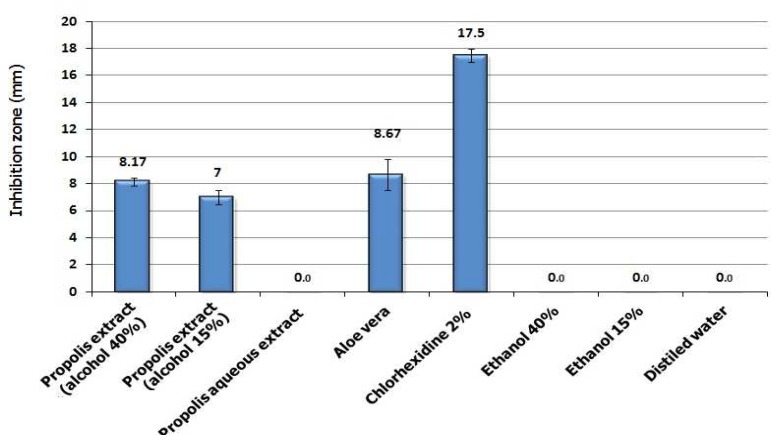
A comparison between Aloe vera and chlorohexidine 2% antibacterial activity against 3 test microorganisms using disk diffusion test. The label numbers are the mean of inhibition zone for three replicate disks

Propolis hydroalcoholic extract (with 15 and 40% ethanol) and Aloe vera gel showed antibacterial effect with no significant difference among them. However, no inhibition zone was observed with propolis aqueous extract (0% ethanol). Chlorhexidine 2% produced significantly higher inhibition zone compared to the other extracts (P< 0.001) (Fig. 1). The Aloe vera gel was less effective than Chlorhexidine 2% not only against *E. faecalis* but also against *S. aureus* and *S. mutans* (Fig. 2).

**Table 1:** The quality control assays on propolis sample and its extracts

**Result (unit)**	**Conducted assay**
brown	Sample color
26.2 (%)	Total polyphenol content
Negative (cfu/g)	E. coli growth
Negative (cfu/g)	*Staphylococcus aureus* growth
Negative (cfu/g)	Pseudomonas Sp. growth
Negative (cfu/g)	Aspergillus growth
55.8 (%)	Dried mass
16.3 (%)	Total carbohydrate content
0.5 (%)	Total protein content
Positive	Free amino acid (detected by TLC)
Positive	Free sugars (4 and 5 carbon detected by TLC)
2.35 (%)	Insoluble substances in 10% alcohol
2.87 (%)	Reduced sugar
0.1 (%)	Dry substance of saturated aqueous extract (0% ethanol)
0.5 (%)	Dry substance of propolis hydroalcoholic extract (40% ethanol)
0.3 (%)	Dry substance of propolis hydroalcoholic extract (15% ethanol)

**Table 2:** The physicochemical analysis of Aloe vera sample

Color	Colorless
pH	4.45
Density	0.9739 grml **(**g/ml)
Dry weight	0.9 %
Ash weight	0.29%
Viscosity	2.0575 **)**cP(
Glucomannan	0.049%
Carbohydrate	0.43%

**Microdilution test**

The MIC results for propolis hydroalcoholic extracts, Aloe vera gel and Chlorhexidine 2% solution have been presented below (Table 3).The propolis aqueous extract (0% ethanol) did not show any inhibition in microdilution test (MIC> propolis solubility). Chlorhexidine showed the lowest MIC (2 µg/ml) compared to the other tested materials. In addition to *E.* *faecalis*, Aloe vera showed antibacterial activity against two gram positive cocci (*S. aureus, S. mutans*) in this test (Table 3).

**Direct contact test**

**Fig. 3:**
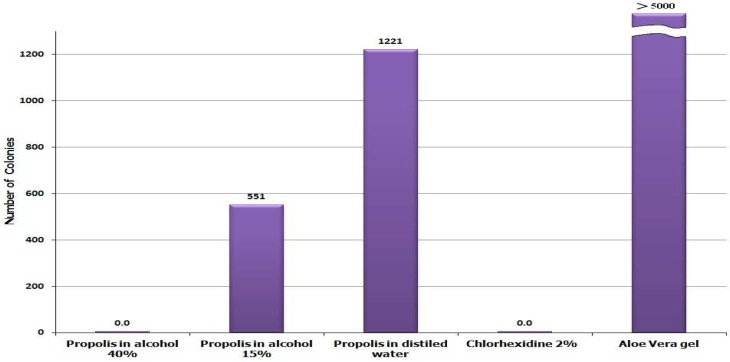
The number of grown colonies of *E. faecalis *after 24 hour contact with propolis hydroalcoholic extracts, Aloe vera, and Chlorohexidine in direct contact test

The number of colonies of bacteria grown after 24 hours is shown in fig. 3. The hydroalcoholic extract of propolis with 40% alcohol showed significant antibacterial effect against *E. faecalis* (similar to Chlorhexidine 2% solution). The aqueous extract of propolis showed a lesser extent in this antibacterial effect. However, Aloe vera showed no antibacterial effect in this method and the resulting colonies were practically uncountable same as the negative controls (because of countless resulting colonies, the negative controls are not depicted in this figure).

## Discussion

In this study, we showed that Aloe vera gel and propolis ethanolic extracts have antibacterial activity against *E. faecalis* in *in vitro*. However, both these naturally available substances showed lower potency compared to Chlorhexidine in either disk diffusion and microdilution assays (Table 3, Fig. 1, 2). On the other hand, propolis ethanolic extract showed high antibacterial activity against *E. faecalis* comparable to that of Chlorhexidine in direct contact test (Fig. 3) which signifies the importance of solubility issue. Some gram positive bacteria such as *E. faecalis *resist the cleaning and shaping of root canal, and potentially can lead to endodontic failure (13-15).

Aloe vera gel and propolis are two naturally occurring substances which have been long used in the treatment of inflammation and infectious diseases of the mouth (13, 16-17). The physicochemical assays conducted on both Aloe vera and propolis samples confirm their standard characteristics (Tables 1, 2). Since the solubility of propolis components in alcohol is different, the concentration of ethanol used for extraction is critical. The dry weight of each propolis alcoholic extract is correlated to its ethanol concentration (Table 2). In this study, we used high speed centrifugation following filtration to omit any dispersed solid material off the extract. Colloidal particles in the extract might exert direct antibacterial effects. The noticeable difference in antibacterial activity results obtained by the three test procedures, especially with propolis aqueous extract, indicates that ethanol soluble constituents of propolis are responsible for its antibacterial effect (8, 18). These components show quite high antibacterial activity in direct contact test against *E. faecalis *(Fig. 3). 

The anti-microbial effect of hydroalcoholic extracts of propolis in disk diffusion was less than that in microdilution, this issue might be aroused by low diffusion ability of alcohol soluble components in agar. On the other hand, since Aloe vera gel is aqueous, no such a difference was observable between its microdilution and its disk diffusion test (Fig. 1, Table 3)

In direct contact test the microorganism gets in touch with the surface of the dried material directly, hence, there is no problem with insolubility of antimicrobial components. For this reason, the aqueous extract of propolis, which contains the least amount of ethanol soluble antimicrobial components, only shows its weak antibacterial activity in direct contact test (Fig. 3).

**Table 3:** MIC and MBC in hydroalcoholic extract of propolis, Aleo vera and Chlorohexidine 2 % using the microdilution test on *E. faecalis*

**MBC(µg/ml)**	**MIC(µg/ml)**	**Groups**
625	313	Propolos hydroalcoholic extract (40% ethanol)
1500	750	Propolos hydroalcoholic extract (15% ethanol)
NA	NA	Propolis aqueous extract (0% ethanol)
4500 NA ^(^^1^^)^ 4500 ^(^^2^^)^	2250 4500 ^(^^1^^)^ 2250 ^(^^2^^)^	Aloe vera
4	2	Chlorohexidine 2%
NA	NA	Ethanol40 %
NA	NA	Ethanol 15%
NA	NA	Distilled water

NA: without antibacterial inhibitory effect ^(^^1^^)^ The test microorganism was *S. aureous* ^(^^2^^)^ The test microorganism was *S.* *mutans*

These substances have low solubility in water but they are highly soluble in ethanol. Some components in propolis, which have been suggested as its active agents, include flavonoids, phenolic and aromatic compounds like caffeic acid (19). Our results are in concordance with a study conducted by Mattigatti et al. (2012) who investigated the effects of propolis on three microorganisms (*E. faecalis,* *S. aureus* and *Candida* *albicans*) using agar diffusion test (20). They have shown (same to our results) that Chlorhexidine along with MTAD® (a mixture of tetracycline, citric acid and a detergent) has superior activity against the tested micro-organisms.

On the other hand, Aloe vera gel which showed weak antibacterial activity in disk diffusion and microdilution tests, failed to show any activity in direct contact test (Fig. 3). This might be the result of low concentration of its antibacterial components compared to nutrient polysaccharides which could prevent the microorganism to be fully in touch with the Aloe vera active components.

Aloe vera’s pharmacotherapeutic and cosmetic properties have been studied since long time ago (16-17). However, studies about its antibacterial effect on *E. faecalis *and its comparison to intra canal drug like Chlorhexidine 2% has not yet been done. The leaf of Aloe vera contains some active substances like acemanan, anthraquinone, anthracine, cinnamonic acid with anti inflamma-tory/antimicrobial properties (17, 21).

As a comparison between Aloe vera and propolis, the antimicrobial effect of Aloe vera gel in microdilution was less than hydroalcoholic extracts of propolis and its obtained MIC on all tested microorganisms (*E. feaclais*, *S. aureus* and *S.* *mutans*) was more than 2250 µg/ml. Recently, conducted studies with other test organisms or methods of antibacterial activity assyas, have shown similar results in our study. In the study by Anuj Bhardwaj et al. in 2012, the antimicrobial effect of some natural extracts and Aloe vera with Chlorhexidine 2% on *E.* *faecalis* was compared which similar to the present study (22-23).

## Conclusion

Aloe vera gel has mild antibacterial effect against *E. faecalis,* *S. aureus* and *S. mutans*. It seems that Aloe vera gel has low antibacterial potency compared to propolis, hence its subfractionation may be a good choice to make a better antibacterial compound for root canal treatments. On the other hand, the hydroalcoholic extract of propolis could be a good anti-microbial agent against *E. faecalis *especially following direct contact to this germ. Both tested natural substances have less antibacterial activity compared to Chlorhexidine , however their potency could be significantly increased by improvement in the extraction techniques. This could potentially lead to root canal antibacterials with fewer side effects.
